# *Equisetum hyemale*-derived unprecedented bioactive composite for hard and soft tissues engineering

**DOI:** 10.1038/s41598-022-17626-w

**Published:** 2022-08-04

**Authors:** Rosangela Maria Ferreira da Costa e Silva, Ivana Márcia Alves Diniz, Natália Aparecida Gomes, Guilherme Jorge Brigolini Silva, José Maria da Fonte Ferreira, Rubens Lucas de Freitas Filho, Erico Tadeu Fraga Freitas, Darliane Aparecida Martins, Rosana Zacarias Domingues, Ângela Leão Andrade

**Affiliations:** 1 Universidade Federal da Fronteira Sul, Campus Realeza-PR, Realeza, Paraná, 85770-000 Brazil; 2 Restorative Dentistry Department, Universidade Federal de Minas Gerais, Belo Horizonte, Minas Gerais 31270-901 Brazil; 3 Departamento de Engenharia Civil, Universidade Federal de Ouro Preto, Minas Gerais, Ouro Preto, 35400-000 Brazil; 4 Department of Materials and Ceramic Engineering, CICECO -Aveiro Institute of Materials, University of Aveiro, 3810-193 Aveiro, Portugal; 5 Departamento de Química, Universidade Federal de Minas Gerais, Belo Horizonte, Minas Gerais 31270-901 Brazil; 6 Centro de Microscopia, Universidade Federal de Minas Gerais, Belo Horizonte, Minas Gerais 31270-901 Brazil; 7 Instituto Federal Do Paraná, Umuarama, Paraná, 87507-014 Brazil; 8 Departamento de Química, Universidade Federal de Ouro Preto, Ouro Preto, Minas Gerais 35400-000 Brazil

**Keywords:** Medical research, Chemistry, Engineering, Materials science

## Abstract

Although Bioactive Glasses (BGs) have been progressively optimized, their preparation often still involves the use of toxic reagents and high calcination temperatures to remove organic solvents. In the present work, these synthesis related drawbacks were overcome by treating the ashes from the *Equisetum hyemale* plant in an ethanol/water solution to develop a bioactive composite [glass/carbon (BG-Carb)]. The BG-Carb was characterized by scanning electron microscopy, and transmission electron microscopy; and its chemical composition was assessed by inductively coupled plasma-optical emission spectroscopy. Brunauer–Emmett–Teller gas adsorption analysis showed a specific surface area of 121 m^2^ g^−1^. The formation of hydroxyapatite (HA) surface layer in vitro was confirmed by Fourier-transform infrared spectroscopy analysis before and after immersion in simulated body fluid (SBF) solution. The Rietveld refinement of the XRD patterns and selected area electron diffraction analyses confirmed HA in the sample even before immersing it in SBF solution. However, stronger evidences of the presence of HA were observed after immersion in SBF solution due to the surface mineralization. The BG-Carb samples showed no cytotoxicity on MC3T3-E1 cells and osteo-differentiation capacity similar to the positive control. Altogether, the BG-Carb material data reveals a promising plant waste-based candidate for hard and soft tissue engineering.

## Introduction

Bone grafting using silica compounds was developed by Larry L. Hench in the late 1960s^1,2^. Besides silica as main compound, the different bioactive glass formulations also often include variable amounts of alkali (Na_2_O up to 25% w/w), alkali earth (CaO up to 25% w/w), and P_2_O_5_ (up to 6% w/w)^3–5^. Bioactive glasses are so named because of their ability to bind to hard and soft tissues. Bonding to living tissues occurs through a HA layer formed on the glass surface^3,6^. Today, several combinations of these elements are commercially available as grafting materials for bone regeneration^7^. However, the repair of critical-sized bone defects requires smart biomaterials combining the capacity of promoting angiogenesis, osteogenesis, with antibacterial activity^8,9^ and low fabrication costs. The production of bioactive glasses, or their composites, is expensive and has to comply with high requirements in terms of reagent purity, and strict control of the production processes^10^. In addition, the most common production processes have some limitations. For example, the melt-quenching method requires high temperatures. The sol–gel synthesis method involves lower temperatures, but the most used source of silica, the tetraethyl orthosilicate (TEOS), is a toxic reagent^10–17^. Therefore, novel approaches are required to overcome these shortcomings.

In contrast, safe biogenic silicon or silica derivatives can be obtained from natural sources such as rice husks, corn cobs, corn straw, rice, and sugarcane bagasse, as well as from genus *Equisetum* plants^17–21^. About 30 species of genus *Equisetum* plants can be found in all continents, being popularly known as horsetail plants. They can also be cultivated and are easy to propagate in calcareous and moist soil^22,23^, bringing ecological issues such as cultivation in areas of difficult access and sustainable farming or extractives.

Relative to dry mass, *Equisetum* genus plants may contain silica from 0.1 to 25%, carbon in up to 40%, besides other elements such as sodium, calcium, potassium, magnesium, phosphorus, iron and zinc in high percentages^21,23–26^. The elements carbon, sodium, potassium, silicon, calcium, magnesium, phosphorus, iron, manganese, copper, and zinc are components of human organisms, playing a diversity of biological functions^7,10,27,28^. Carbon-related characteristics such as high specific surface area and porosity, are both desirable in bioactive glass scaffolds for inducing osteointegration and bone regeneration^7,27–29^. Silicon is reported as an "essential mineral" in the formation of collagen, cartilages, and bone tissues^30^. Silicon contributes to the regulation of bone absorption and contributes in the early stage of bone calcification and densification^31^. Calcium is the main component of human bones in the form of HA [Ca_10_(PO_4_)_6_(OH)_2_] phase (> 99% Ca), and its main function is to maintain the skeletal framework. Ceramics or metallic implants functionalized with HA or with coatings of other calcium phosphate phases exhibit improved osteoconductive and osteoinductive proprieties^32^. Magnesium is also an integral part of the bone structure. Copper ions exert an antibacterial action, and are osteoinductive, stimulating the proliferation and the activity of endothelial cells and osteoblasts^33^. Manganese is reported to act in the maintenance of the bone structure, regulating bone metabolism, and local treatment of ion manganese accelerates fracture healing^34^. Zinc ions are reported to stimulate osteoblast bone formation, increase alkaline phosphatase activity, and inhibit osteoclast differentiation^35–37^. This is to underline that the presence of these therapeutic ions in the biogenic plant derivative bone graft materials may be regarded as potential benefits in terms of bone regeneration performance.

On the other hand, the pertinency of purification processes aiming at eliminating all ions other than silicon from the plant derivative silica is questionable, especially when further considering that such processes involve many steps and demand a lot of time and energy like burning, alkalization (pH > 10) followed by the addition of acid^18,24–26^. The process for increasing the purity level of silica includes steps prior to burning and/or base solubilization such as leaching with hydrochloric acid or citric acid to remove the Na, K, Mg, Ca, Fe, Zn ions; ammonia solubilization of the persistent ions Na, Cl, P, and S; drying and burning at high temperatures; and crushing for several hours^17,18,24,26,38^. However, the majority of the elements or ions present in the *E. hyemale* waste are often intentionally incorporated in bioactive glasses or in the simulated body fluid (SBF) solutions used to assess their biomineralization ability^39^. Therefore, these elements and ions are desired and the steps for eliminating them are unnecessary.

In this paper, considering the prior knowledge on the *E. hyemale* composition, we hypothesized that the entire composition of the *Equisetum* waste could have the potential to produce a low-cost biocompatible carbon/bioactive glass composite of high efficiency as bone substitute. Ultimately, our study suggests a new ecological route for extracting all the potential benefits from a natural sustainable resource plant to develop a promising high added value biomaterial.

## Experimental

### Materials and methods

The following commercial grade reagents were used as received from the corresponding suppliers: ethanol 96 GL (Êxodo), hydrochloric acid PA (Synth). Deionized water (DI water) was obtained at the lab by using a water deionization system (Permution, DE1800).

### Preparation of the BG-Carb composite

*Equisetum hyemale* plants were harvested, washed with deionized water, and dried at room temperature for two days. Samples of plants were deposited in the herbarium unit of the herbarium UPCB of Universidade Federal do Paraná with number 99428, have been digitized and are available for viewing at available in http://upcb.jbrj.gov.br/v2/consulta.php. The authors confirm that the use of plants in the present study complies with international, national, and/or institutional guidelines. The research with *equisetum hyemale* were indexed in the Sistema Nacional de Gestão do Patrimônio Genético e do Conhecimento Tradicional Associado (SISGEN, Brazil) according to the certificate ADBDCBE.

The dried plants were placed in a muffle at 300 °C for 2 h. The plant ash was crushed and solubilized in a 50:50 v/v ethanol (E): deionized water (DI) solution (30 g ash for 70 mL of the E:DI water solution). The resulting alkaline suspension (pH = 11) was left under stirring for 2 h at 80 °C, and then filtered through filter paper. The ash was dispersed again in the E:DI water solution for 15 min at 80 °C and filtered. This washing process was repeated for three times for reducing the concentrations of sodium, potassium and any organic substances that might have been formed in the burning in the plant, and concomitantly concentrate the final material in silica, calcium, and phosphorus. The pH of the resulting suspension was corrected to pH 7.0 with PA hydrochloric acid. The solid particles were filtered, dried, and called BG-Carb.

### Characterization

Mineral contents of the ashes of the plant *Equisetum hyemale* and of the BG-Carb were determined by plasma atomic emission spectrometry (Perkin Elmer Optima 5200 DV)^40,41^. The total carbon was determined by the combustion loss method in muffle (Quimis, 0318M24). Fourier-transform infrared spectroscopy (FTIR) was used to detect the hydroxyapatite functional groups in the samples using a Perkin Elmer FT-IR GX model instrument. The solid samples were homogeneously mixed with KBr in a 1:100 w/w ratio and disc-pressed. The FTIR spectra were recorded in transmittance mode at a resolution of 4 cm^−1^ from 4000 to 400 cm^−1^ and 32 scans per sample. The crystalline phases of the bulk samples were identified using a Bruker D2 Phaser 2nd Generation powder X-Ray diffractometer (Bruker Company, USA) with setup routine of CuKα radiation (1.54184 A), operating at 30 kV and 10 mA, scanning range from 7 to 70 $^\circ$ and a step size of 0.02 $^\circ$, 1 s per step. The phase identification was achieved using Jade + software (Materials Data, Inc.). The Rietveld refinement was carried out with the FULLPROF SUITE 2019 program (available at https://www.ill.eu/sites/fullprof/). All of the standard cif files used for phase identification were provided by Inorganic Crystal Structure Database (ICSD). Transmission electron microscopy (TEM), selected area electron diffraction (SAED), and energy dispersive X-ray spectroscopy (EDS) were performed in a thermionic LaB6 FEI microscope Tecnai G2-20 SuperTwin, operated at 200 kV, coupled with an Oxford Xplore silicon-drift detector (SSD-EDS). The TEM samples were prepared by placing a drop of each diluted sample in different holey carbon-coated TEM Cu-grids (300 mesh) (Electron Microscopy Sciences) and let drying until analysis. The TEM sample preparation and analysis were performed in the Center of Microscopy at the Universidade Federal de Minas Gerais. The morphology of the samples was assessed by conventional TEM and scanning electron microscopy (SEM). For SEM a Tescan Vega3 microscope coupled with EDS was used, with an acceleration voltage of 5.0 kV and 15 kV. The samples were coated with a thin Au layer. The crystallographic phase was evaluated by SAED, and the elemental composition estimated by EDS. Nitrogen adsorption/desorption analyzes were performed in samples previously degassed at 150 $^\circ$C for 12 h under vacuum conditions using an Autosorb iQ equipment (Quantachrome Instruments, USA) at − 196 $^\circ$C in the relative pressure range of 0.005–1.0. The amount of material used was about 300 mg and the analysis time was 8 h. The surface area of each material was estimated by the BET (Brunauer, Emmett, Teller) method, and the pore size distribution was estimated by the BJH (Barrett-Joyner-Halenda) method. The acquisition of experimental data and data processing were obtained using ASiQwin version 5.21 software. From the N_2_ adsorption isotherm, the specific surface area was calculated by the BET method within the pressures range of 0.05–0.30. The volume and area of these micropores were calculated using the t-plot method.

### Cell culture

MC3T3-E1 (ATCC Subclone 4 CRL-2593) pre-osteoblastic cells were purchased from ATCC (Manassas, VA) and grown in regular medium containing DMEM (Dulbecco’s Modified Eagle's Medium) supplemented with 10% fetal bovine serum (FBS) and 1% of antibiotic (100 IU/ml penicillin, 100 µg/ml streptomycin) (Gibco, Thermo Fisher, Waltham, USA). The cells were kept at 37 $^\circ$C in a humid atmosphere containing 5% CO_2_. Culture media were replaced every 2–3 days.

### Cell viability/proliferation analyses

A 1% (w/v) suspension of the BG-Carb composite was prepared in cell culture medium according to Tsikou *et. al.* and incubated for 24 h, at 37 $^\circ$C, prior to the experiments^42^. Cells were plated at 2 × 10^4^ cells/well in 48-well microplates 24 h before the contact with the biomaterial. Cell proliferation was analyzed at 48 and 96 h using the MTT assay [3-(4,5-Dimethylthiazol-2-yl)-2,5-Diphenyltetrazolium Bromide)] (Invitrogen, Thermo Fisher, Waltham, USA)], according to the manufacturer's recommendations. Cells cultured under ideal conditions were used as controls. Briefly, a 5 mg/mL MTT suspension was prepared and added to the cell cultures for 3 h in the dark at 37 $^\circ$C, in a humid atmosphere containing 5% CO_2_. Then, formazan crystals were dissolved with 100 μL in dimethyl sulfoxide in each well (Sigma Aldrich, St. Louis, USA). The absorbance was determined by optical density in a spectrophotometer (Cytation 5 Multiplate Image Reader, software Gen 5 Image 3.3, Biotek Winooski, USA) with 540 nm filter.

### Cell differentiation analysis

For the cell osteo-differentiation assay, cells were plated at 5 × 10^4^ cells/well in 48-well microplates. BG-Carb-conditioned medium was then prepared and added to the cells for 14 days. Cells grown under osteogenic/inductive media containing DMEM supplemented with 1% of antibiotic (100 IU/mL penicillin, 100 µg/mL streptomycin), 10% FBS (all from Gibco), 180 mM KH_2_PO_4_, and 10^–8^ µM of dexamethasone (both from Sigma-Aldrich) served as positive controls. Medium was changed every 2–3 days throughout the experiments.

### Alizarin red staining (ARS)

The formation of mineralized nodules was detected by using the ARS. Cell cultures were washed twice in PBS, fixed with 75% isopropanol, rehydrated in distilled water, and stained with alizarin red (Sigma-Aldrich) 1%, pH 4.2, 5 min. The cultures were washed three times in PBS and left dry. The wells were photographed using a multimode reader and a 10 × objective (Cytation 5, Biotek). For quantitative analysis, a 10% aqueous acetic acid solution (v/v) and methanol (4:1 v/v) was added to the wells for 30 min for the dissolution of mineralized nodules. The absorbance was determined by optical density using the 490 nm filter.

### Statistical analysis

Data were analyzed using GraphPad Prism 9 software (GraphPad, San Diego, USA). Mann–Whitney test was used to find differences between groups. In all cases, *p* < 0.05 was considered statistically significant.

## Results and discussion

### Composition and FTIR spectra of ashes of the plant *Equisetum hyemale* and BG-Carb composite

**Figure 1:**
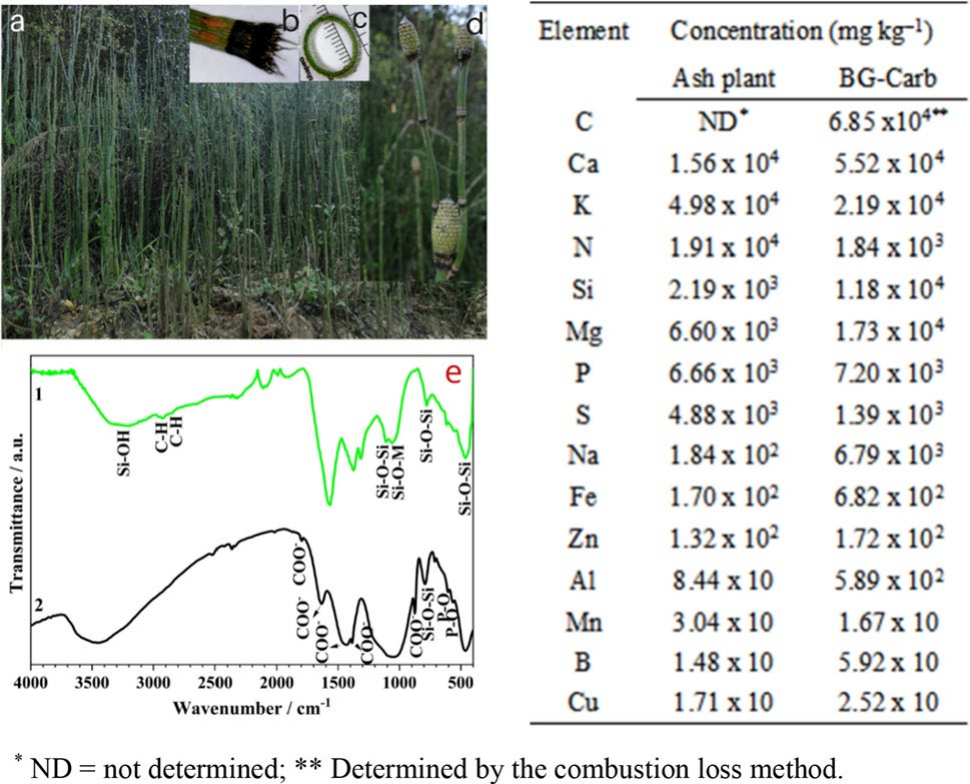
Images of *Equisetum hyemale: *(**a**) *plants *in situ*; *(**b**) *node and leaves; *(**c**)* stem cross section; *(**d**)* strobilus*; (**e**) FTIR spectra of the: (1) *Equisetum hyemale* plant ash, and (2) BG-Carb composite; Concentrations (mg kg^−1^, dry weight) of elements in *Equisetum hyemale* plant ash and in the BG-Carb composite. *ND = not determined; **Determined by the combustion loss method.

The *Equisetum hyemale* plant and its details are shown in Fig. 1 (left), while the elemental compositions of the *Equisetum hyemale* plant ash and of the BG-Carb composite are shown on its right-hand side*.*

The composition of the plant ash is in good agreement with related data described in the literature^23^ and corroborating with thermal analysis (Fig. S1). It is interesting noting that most of the identified components (Na, K, Mg, Ca, HCO_3_^–^, HPO_4_^2–^, SO_4_^2–^) are also present in the composition of blood plasma (or in the simulated body fluid commonly used to study the biomineralization of bioactive glass, Table S1). This turns the material surface friendly, favoring the formation of HA^39^. Furthermore, trace elements such as Mg, Fe, B, Mn, Se, Zn, Co, Cr, and Cu, may confer therapeutic and regenerative functions to the resulting biomaterial. Besides enhancing the mechanical strength and improving the bioactivity and the control over the degradation rate, these trace elements may also provide antibacterial action and induce angiogenesis^33,43–48^. The presence of carbon can enhance the surface area and decrease the density of the scaffold structure, features that favor the bioactivity of bone graft substitutes^7,27,28^.

Figure 1e compares the FTIR spectra of the ash of *Equisetum* and of the BG-Carb composite. The FTIR spectrum of the ash (spectrum (1)) exhibits bands typical of amorphous and crystalline silica. The broad band observed in the range of 3100–3800 cm^−1^ is due to the O–H bond vibrations, being a consequence the adsorbed molecular water in the surfaces of silica and activated carbon. The bands at 3422 cm^−1^ and between 1393 and 1097 cm^–1^ may indicate the N–H group in form of –CO–NH–, showing the existence of the nitrogen functional groups^49^.

The BG-Carb spectrum (2) shows typical bands of the *Equisetum* ash and other bands. The doublet bands at about 2923 and 2851 cm^−1^ are related to the C–H stretching, confirming the presence of the remaining carbonized lignin structures in the plant ash. The bands at 1630 and 1384 cm^−1^ correspond to the stretching modes of COO^–^ group from the salicylic acid in the activated carbon^49,50^. The bands in the range of 1800 to 1650 cm^−1^, at 1460, 881 and that at 712 cm^−1^, corresponds to the bending and stretching vibrations of CO_3_^−2^^51^. Bands around 1108 cm^−1^, 780 cm^−1^ and 460 cm^−1^ are characteristics of Si–O–Si stretching, rocking and bending modes, respectively^52^. The band at 1051 cm^−1^ is commonly observed in the presence of Si–O–M for the metals (M) K, Na and Mg^7,23^. The FTIR spectrum of the BG-Carb presents also bands at around 566 and 604 cm^−1^, characteristic of PO_4_ groups from HA^28^.

### BG-Carb composite characterization

**Figure 2:**
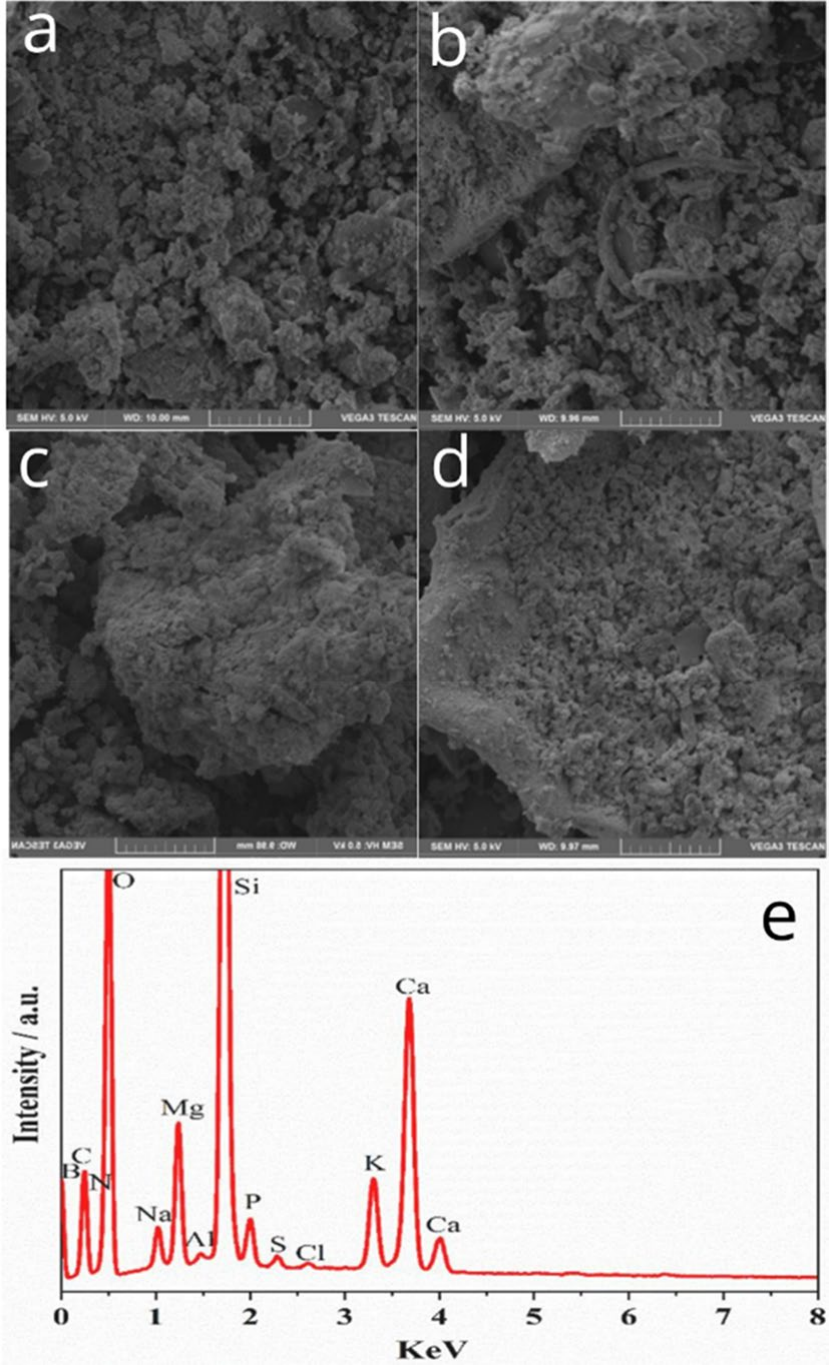
SEM micrographs of BG-Carb composite: (**a**) before, and after immersion in SBF for (**b**) 24 h, (**c**) 48 h, and (**d**) 72 h; and EDS-SEM (**e**) spectrum, for BG-Carb before immersion in SBF.

The SEM images of the BG-Carb composite before and after being immersed in SBF for different time periods are displayed in Fig. 2. The morphological features reveal the presence of some irregularly shaped particles, more evident before and after short-immersion time periods. The surface tends to become more regular with increasing the immersion time, as a consequence of the surface modifications occurring at the solid–liquid interface derived from dissolution–precipitation reactions that lead to a gradual enrichment of the surface in hydroxyapatite. The EDS spectrum of the sample BG-Carb shown in Fig. 2e agrees well with the composition obtained by ICP-OES (Fig. 1).

**Figure 3:**
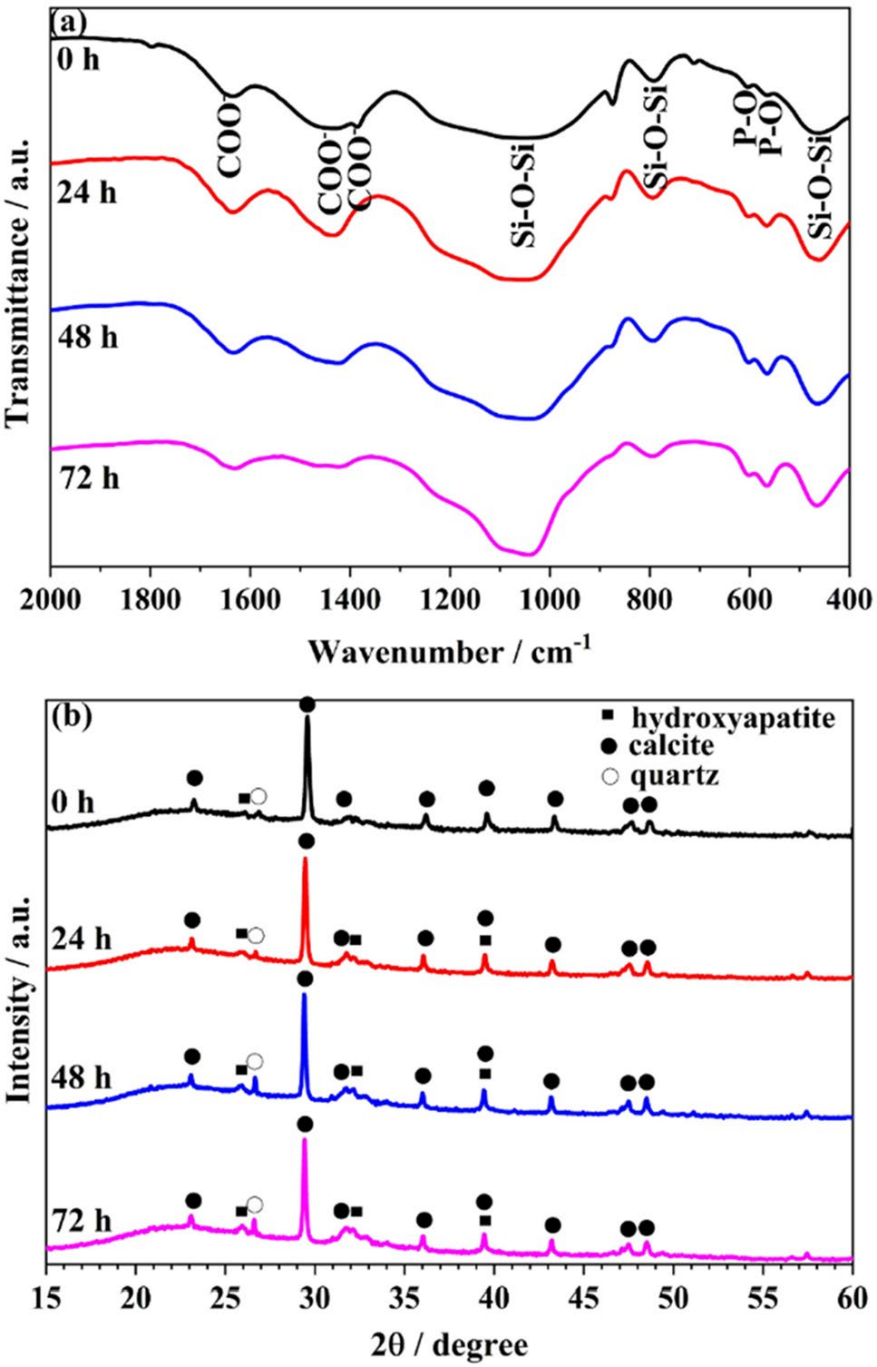
(**a**) FTIR spectra, and (**b**) X-ray diffraction patterns, of BG-Carb composite samples before (0 h), and after immersion in SBF for the different time periods indicated (24 h, 48 h, and 72 h).

The FTIR spectra of the composite BG-Carb composite samples immersed in SBF at the time points: 0 h, 24 h, 48 h, and 72 h are displayed in Fig. 3. Noticeable increases in intensity of the bands at around 566 and 604 cm^−1^, characteristic of PO_4_ groups from HA can be seen. The observed gradual reduction in intensity of the bands at around 1040 cm^−1^, 800 cm^−1^ and 459 cm^−1^, attributed to Si–O–Si, indicates an increasing coverage of silica-rich surface by HA, and/or loss of silicon for the solution^28^. A gradual reduction in intensity of the stretching carbonate bands due to the presence of CO_3_^2−^ (CaCO_3_) at 873 and 1449 cm^−1^ can also be observed^53^. This reduction trend in the intensity of bands characteristic of calcite and the concomitant increase in intensity of bands characteristic HA are supported by X-ray diffraction and SAED results.

The X-ray diffraction (XRD) patterns for the samples BG-Carb, before and after immersing in SBF for 24, 48 and 72 h are shown in Fig. 3. The broad diffuse peak centered at 2θ ≈ 22$^\circ$, attributed to the presence of amorphous silica^54^, indicates that horsetail-derived silica is mainly present as amorphous/low crystalline phase^52^. It can be seen that all X-ray diffractograms indicate the presence of calcite (ICDD # 5–586), hydroxyapatite (ICDD # 1–1008), and quartz (ICDD # 46–1045). It is interesting to note that the hydroxyapatite is present in the sample even before immersion in the SBF solution, as suggested by SEM–EDS and STEM-EDS. This can be explained by the composition of BG-Carb (Fig. 1), which simulates a bioactive glass after immersion in SBF^7,23^.

**Figure 4:**
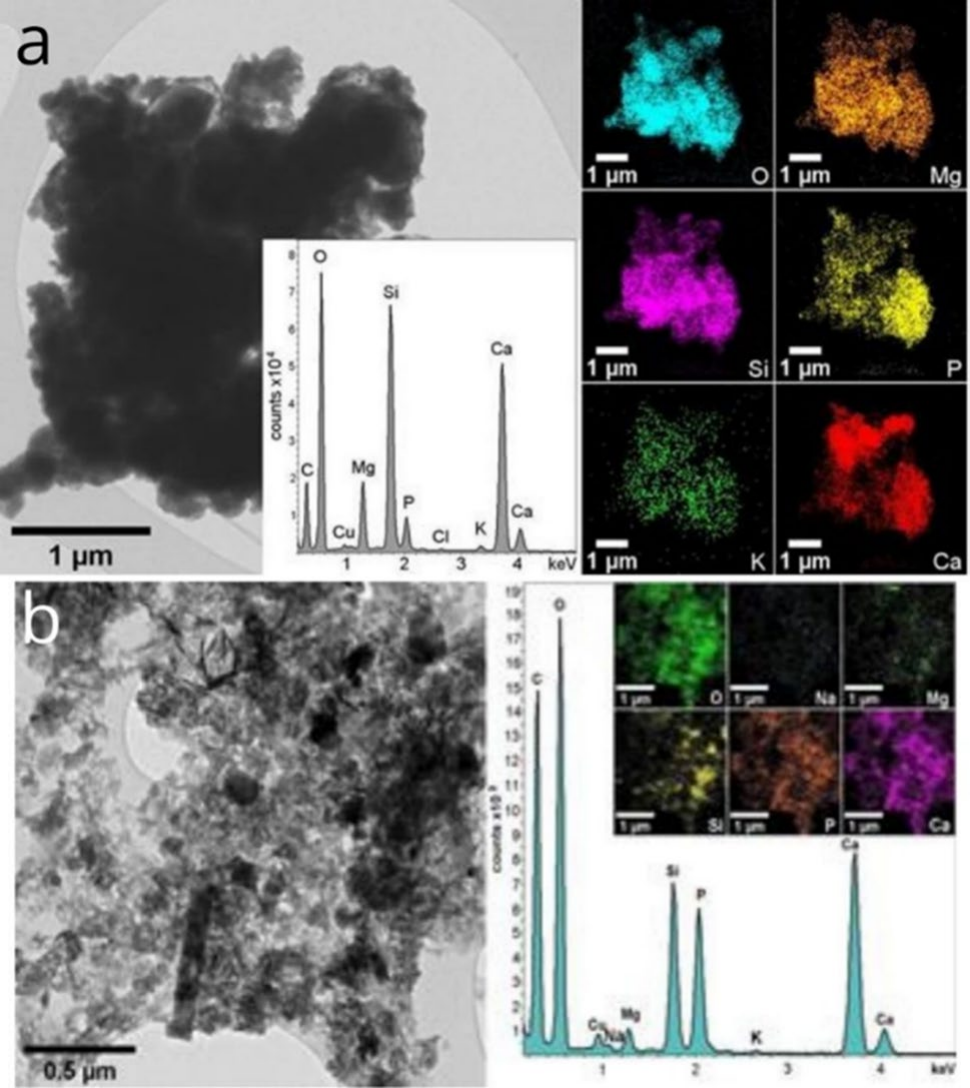
TEM Bright-field images and the EDS data of the: (**a**) BG-Carb sample before immersion in SBF, and (**b**) after 72 h of immersion in SBF. For each sample, the elemental distribution maps are shown on the right-hand side. The Cu signal in EDS spectra arises from the Cu-TEM grid.

**Figure 5:**
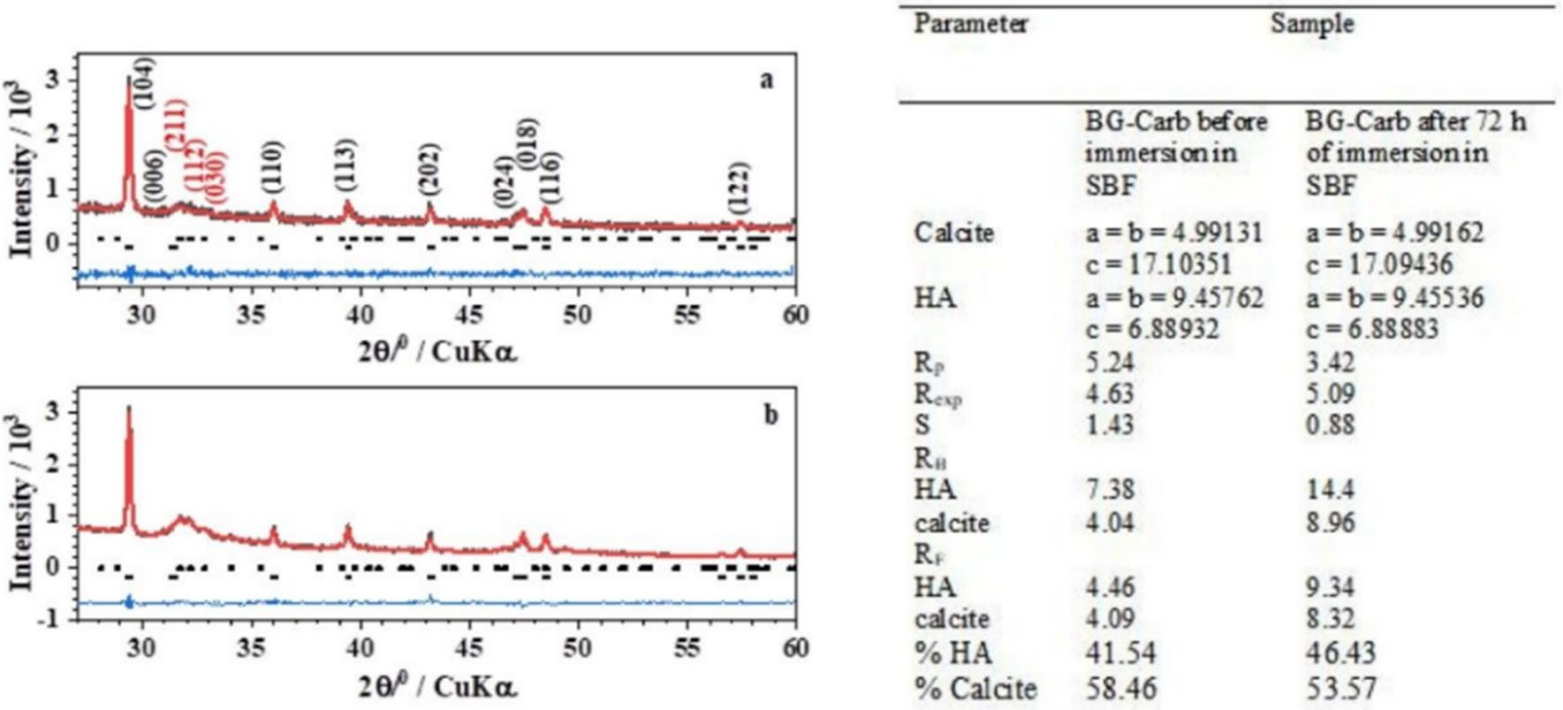
Left: Powder X-ray diffraction patterns (Black) of the BG-Carb samples (**a**) before and (**b**) after immersion in SBF solution for 72 h. The solid (Red) lines correspond to the profiles fitted through Rietveld refinement. The *hkl* indexes in black correspond to calcite phase, and those in red correspond to hydroxyapatite phase. Right: Structural parameters, Rietveld agreement factors, and phase compositions for the samples BG-Carb before and after immersion in SBF for 72 h.

**Figure 6:**
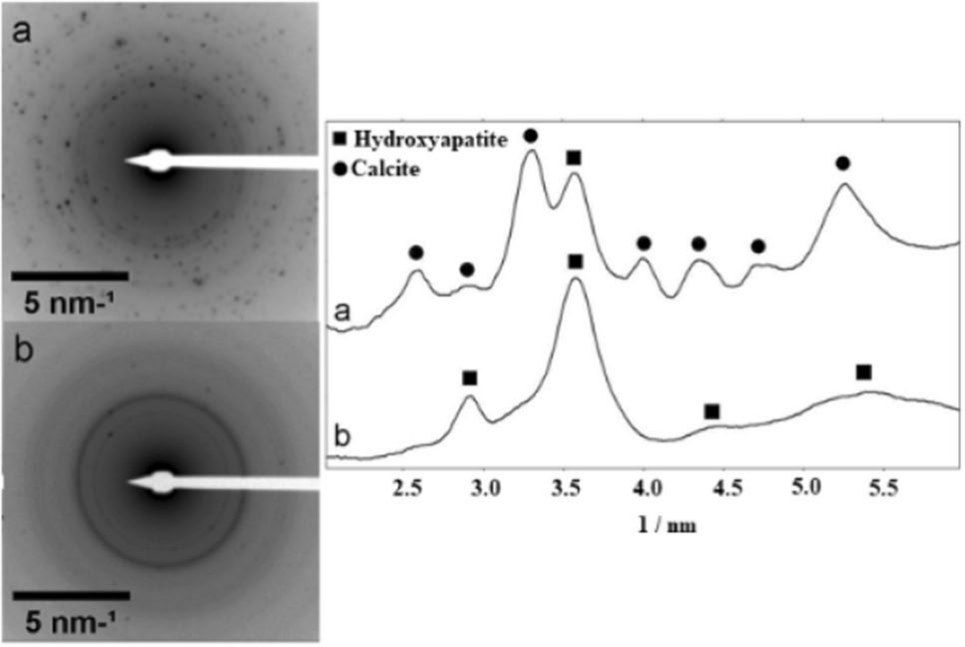
TEM-SAED (**a**) before, and (**b**) after immersion in SBF for 72 h. The profiles of SAED patterns (**a**) and (**b**) are shown with the indication of the reflections of HA and calcite.

Figure 4 shows the TEM images along with the STEM-EDS data from the samples before and after 72 h of contact with the SBF solution. The crystalline particles of the sample were larger before treatment. Particles of calcite and HA up to a few micrometers in size can be discerned from the STEM-EDS maps of after 72 h in SBF solution (Fig. 4). The TEM image of the sample after 72 h of contact with the SBF solution (Fig. 4b) shows reduced particle size. HA seems to be predominant in that sample. Both SEM–EDS and STEM-EDS elemental maps show the presence of likely silicon oxides spread out in the sample. The presence of HA in the samples, before and after immersion in the SBF solution, was also confirmed by Rietveld refinement (Fig. 5, left) and TEM-SAED (Fig. 6).

The structural Rietveld refinement of the powder XRD patterns was performed by fitting pseudo-Voight functions (*R*-3c and *P*63/m space group for calcite and hydroxyapatite, respectively). The quality of the Rietveld refinement was evaluated from the profile factor (R_p_), weighted profile factor (R_exp_), goodness of fit indicator (S), Bragg Factor (R_B_), and crystallographic RF-factor (R_F_) parameters. The data are showed in Fig. 6.

TEM-SAED patterns (Fig. 6) show the presence of calcite and HA in the sample, even before being immersed in SBF. After 72 h of contact with the SBF solution, HA becomes the predominant phase. These STEM-EDS observations are supported by the results of Rietveld refinement (Fig. 5, left). The SAED rings of the sample after 72 h of immersion (Fig. 6b) are more homogeneous in comparison to those of the non-immersed sample (Fig. 6a). These features suggest that an apparent reduction of the crystallite size has occurred along the immersion period, corroborating the morphological analysis (Fig. 2). This can be attributed to the partial dissolution of the original sample in the media and, especially, to its surface coverage with a low crystallinity HA layer, which tends to mask the crystalline features of the underlying material.

**Figure 7:**
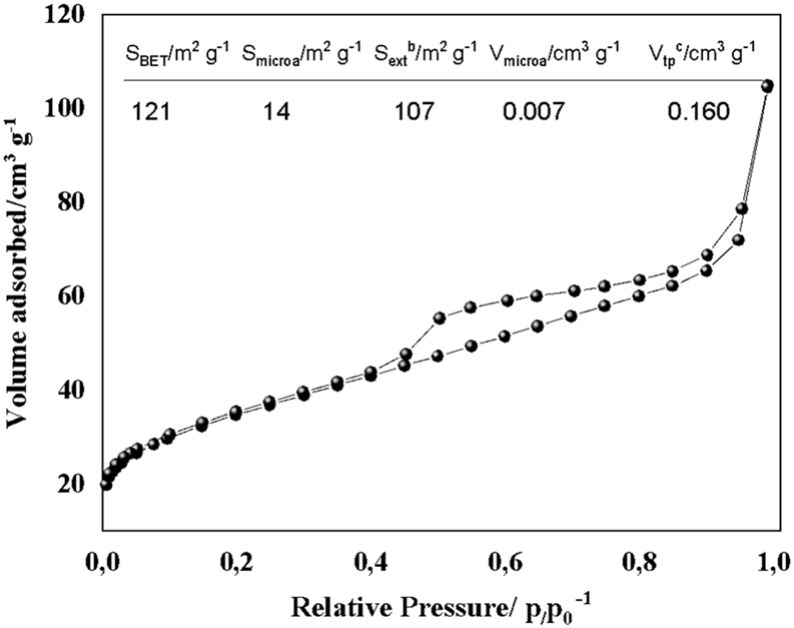
N_2_ adsorption isotherms of the BG-Carb and textural properties of BG-Carb. Where: (**a**) Obtained by t-plot method; (**b**) S_ext_ (external surface) calculated by the subtraction S_BET_—S_micro_; (**c**) Total pore volume obtained at p/p0 = 0.991.

The adsorption isotherm plot of the BG-Carb sample and the related textural properties data determined by the Brunauer–Emmett–Teller (BET) method are shown in Fig. 7. BG-Carb exhibits a specific surface area (SSA) of 121 m^2^ g^−1^, ~ 3.7 times higher than that of *Equisetum hyemale* ash described in the literature (33 m^2^ g^−1^). The surface area of BG-Carb is almost twice as large as sintered bioglass with no additional heating steps^7,28,55^. This difference can be attributed to the presence of carbon^56^. Such increase in the exposed surface area is likely to foster bioactivity. Indeed, the work by Jones^7^ revealed that a bioactive glass with a surface area similar to that of BG-Carb has undergone a faster formation of the HA surface layer upon immersion in SBF. It is also known that high surface areas favor the bonding between the graft and the living tissues after implantation through the formation of HA in the surface^28,29,45,55^. The BG-Carb isotherm (Fig. 7) is a mixture of type I and II isotherms as classified by IUPAC, corroborating finds reported elsewhere^7^. The corresponding hysteresis loop can be classified as an H^4^ type hysteresis. This type of hysteresis occurs due to the formation of aggregate mesopores. H^4^ loops are often found with aggregated crystals of zeolites, some mesoporous zeolites, and micro-mesoporous carbons.

**Figure 8:**
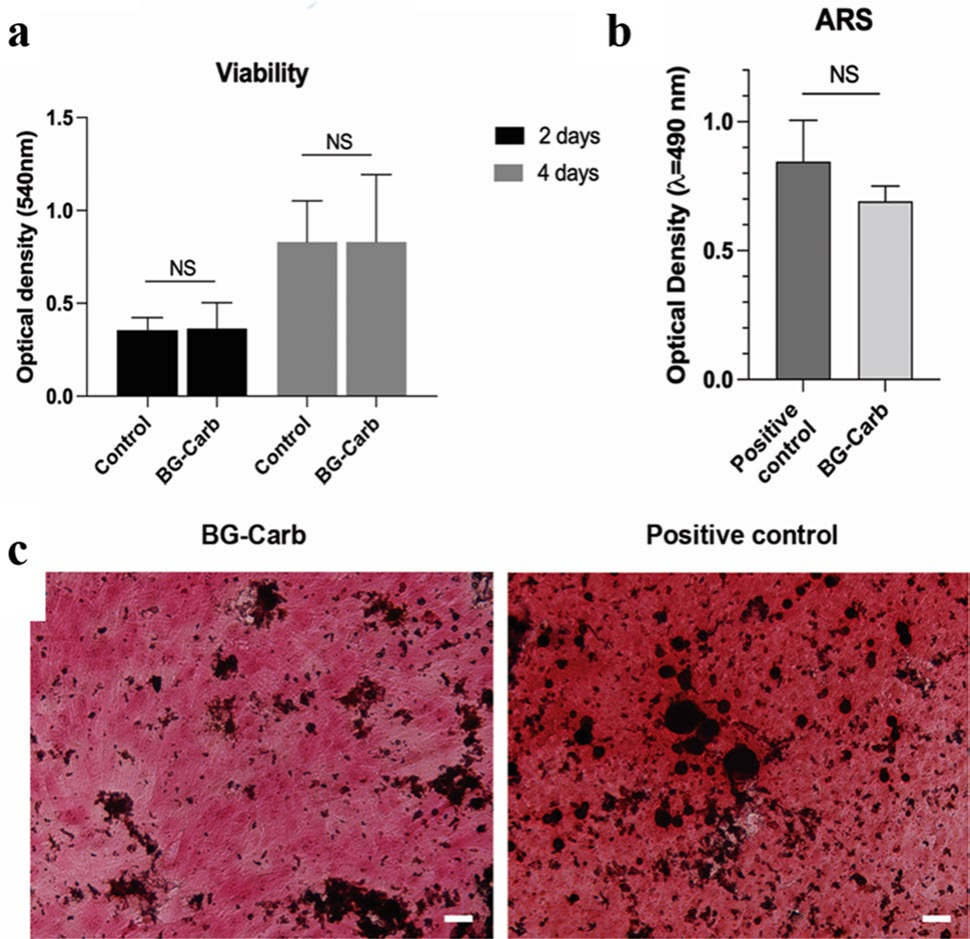
Cytocompatibility of BG-Carb: (**a**) Bar graph showing MC3T3-E1 viability/proliferation upon contact with BG-Carb 1% w/v conditioned medium^29^ for 2 or 4 days; (**b**) Bar graph showing the quantification of mineralized nodule formation upon contact with media conditioned with 1% w/v BG-Carb for 14 days; (**c**) Representative photomicrographs of ARS staining (100x). Data is expressed as mean ± SD (Mann–Whitney test). NS = Not significant. Scale Bar = 20 µm.

BG-Carb tested as a conditioned medium presented cell viability/proliferation similar to the control cultured under ideal conditions during the entire experimental time (Fig. 8). Cells cultured with BG-Carb conditioned medium showed remarkable formation of mineralized nodules, similarly, to the positive control grown under osteogenic inductive medium (Fig. 8b), showing no apparent toxicity on MC3T3-E1 cells^57^.

Figure 8c shows the calcium deposition as a marker of the late stage of osteogenesis. For that, alizarin red stain was used to identify the calcium produced by MC3T3-E1 cells. MC3T3-E1 cells in contact with BG-Carb showed evidence of cell morphology similar to control cells after 14 days. There was no evidence of cellular lysis, and no inhibitory effects on cell growth were detected. The brick red deposits were proportional to the amount of calcium generated by the cells. Cells cultured with BG-Carb conditioned medium showed remarked formation of mineralized nodules (biomineralization), similarly to the positive control grown under osteogenic inductive medium (Fig. 8, right). Therefore, the BG-Carb promotes cell proliferation without affecting the normal functioning of the cells. These data indicate the ability of BG-Carb to form matrix mineralization, which was positively associated with osteoblast proliferation and differentiation^29^.

## Conclusion

A composite material synergistically combining a bioactive glass/carbon (BG-Carb) with unique overall properties was developed in this work by using a simple process. The as obtained biomaterial has a high potential for applications in hard and soft tissues engineering. The results showed that the BG-Carb sample contains hydroxyapatite and ions that simulate human plasma. In addition, it exhibits a larger surface area than those commonly found in bioactive glasses produced by traditional methods. Such characteristics may have been decisive in determining the high level of bioactivity of BG-Carb. Our preliminary biological assessment data indicate that the BG-Carb is cytocompatible, promotes cell proliferation and calcium deposition in the MC3T3-E1 pre-osteoblastic cell cultures. Therefore, the reported results demonstrate that *Equisetum* genus plant is an interesting natural, ecological, and sustainable resource for the preparation of a composite BG-Carb biomaterial through a simple and straightforward method. The as obtained biomaterial exhibits interesting biological properties, making the BG-Carb composite very promising as candidate material for future studies in bone regeneration and tissue engineering applications.

## Supplementary Information

Supplementary Information.

## Data Availability

The datasets generated during and/or analyzed during the current study are available from the corresponding author on reasonable request (R.M.F.C.S).
