# Supplementary material for: Equisetum hyemale-derived unprecedented bioactive composite for hard and soft tissues engineering
Source: Sci Rep. 2022 Aug 4;12:13425. doi: 10.1038/s41598-022-17626-w (PMC9352763; doi:10.1038/s41598-022-17626-w)
Supplement: Supplementary file 1 — Supplementary Information. [file 41598_2022_17626_MOESM1_ESM.docx]

**Supplementary Material**

***Equisetum hyemale*-derived unprecedented bioactive composite for hard and soft tissues engineering**

Rosangela Maria Ferreira da Costa e Silva^1,*,⸸^, Ivana Márcia Alves Diniz^2,⸸^, Natália Aparecida Gomes^2,⸸^, Guilherme Jorge Brigolini Silva^3,⸸^, José Maria da Fonte Ferreira^4,⸸^, Rubens Lucas de Freitas Filho^5,⸸^, Erico Tadeu Fraga Freitas^6,⸸^, Darliane Aparecida Martins^7,⸸^, Rosana Zacarias Domingues^5,⸸^, Ângela Leão Andrade^8,⸸^

^1^Universidade Federal da Fronteira Sul, Campus Realeza-PR, Realeza, Paraná, 85770-000, Brazil.

^2^Departamento de Odontologia Restauradora, Universidade Federal de Minas Gerais, Belo Horizonte, Minas Gerais, 31270-901, Brazil.

^3^Departamento de Engenharia Civil, Universidade Federal de Ouro Preto, Ouro Preto, Minas Gerais, 35400-000, Brazil.

^4^CICECO -Aveiro Institute of Materials, Department of Materials and Ceramic Engineering, University of Aveiro, Aveiro, 3810-193, Portugal.

^5^Departamento de Química, Universidade Federal de Minas Gerais, Belo Horizonte, Minas Gerais, 31270-901, Brazil.

^6^Centro de Microscopia, Universidade Federal de Minas Gerais, Belo Horizonte, Minas Gerais, 31270-901, Brazil.

^7^Instituto Federal do Paraná, Umuarama, Paraná, 87507-014, Brazil.

^8^Departamento de Química, Universidade Federal de Ouro Preto, 35400-000, Ouro Preto, Minas Gerais, Brazil.

^⸸^these authors contributed equally to this work

^*^corresponding. rosangela_ferreirafeliz@yahoo.com.br

Keywords: *Equisetum hyemale*; bioactive glass composite; biomaterials; green bioactive glass composite; bioactive composite.

**Experimental**

The TG and DTG curves were obtained in a Shimadzu Simultaneous TGA/DTA Analyzer DTG-60H equipment. The analyzes were carried out in an alumina crucible and the following furnace settings were used: heating rate of 10 ºC ⋅ .min^−1^, starting from room temperature up to 900 ºC, in an air atmosphere with a flow of 50 mL ⋅ min^−1^. Weights of weighed samples were around 2 to 5 mg.

**Results and discussion**

Table S1: Concentration of ion in blood plasma and SBF solution^6^.


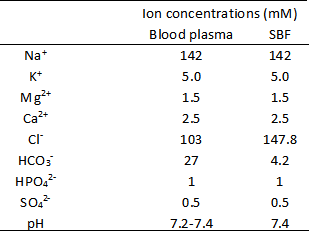


Fig. S1 shows the TGA and DTG graphs of the BG-Carb sample. TGA has enabled to determine its chemical composition in terms of metal oxides and to estimate the carbon loss. The first weight loss (~5 w/w %) is due to water evaporation. A second noticeable weight loss that started near 330 ºC can be attributed to the gradual release of volatile substances, which burn off at higher temperatures, originating the sharp exothermic peal centered at ~431 ºC (DTA, Fig. S2). As temperature increases, the BG-Carb sample underdoes two further significant stepwise weight losses, one between 400−490 ºC, and the other between 490−680ºC, which correspond to the long-lasted and slow degradation process of residues of lignin, hemicellulose and cellulose^58,59^. The non-metals as C and N were completely decomposed up to nearly 660 ºC. The remaining material (80 w/w %) may be assigned to the metal oxides^25^ and SiO_2_ residual corroborating with elemental composition (Fig. 1f).





Fig. S1. TGA and DTG graphs of the BG-Carb.

Fig. S2. DTA graphs of the BG-Carb.
